# Spectral detection of thalassemia: a preliminary study

**DOI:** 10.1186/1423-0127-21-26

**Published:** 2014-03-29

**Authors:** M S AlSalhi, Farjah H AlGahtani, S Devanesan, V Trinka Vijmasi, K Jeyaprakash, Abbas H AlSaeed, V Masilamani

**Affiliations:** 1 Department of Physics and Astronomy, College of Science, King Saud University, P. Box: 2455, Riyadh 11451, Kingdom of Saudi Arabia; 2 Department of Haematology, King Khalid University, Riyadh, Kingdom of Saudi Arabia; 3 Research Chair, Laser Diagnosis of Cancers, King Saud University, Riyadh, Times New Roman; 4 Research and Development Centre, Bharathiar University, Coimbatore, India; 5 Proctor Foundation, University of California, San Francisco, CA, USA; 6 PG and Research Department of Biochemistry, Rajah Serfoji, Govt College, Thanjavur, India; 7 College of Medical Sciences, King Saud University, Riyadh, Kingdom of Saudi Arabia

**Keywords:** Blood plasma, Synchronous fluorescence excitation spectra, red blood cells (RBC), receiver operator curve (ROC), Thalassemia

## Abstract

**Background:**

Thalassemias (Thal) are forms of inherited autosomal recessive blood disorders arising out of mutations in the chromosomes 11 or 16. These disorders lead to poor oxygen delivery to blood vessels and consequent splenomegaly, bone deformities, and shorter life spans. The most common detection methods for Thal are complete blood count (CBC) followed by electrophoresis and molecular diagnosis methods, such as high-performance liquid chromatography (HPLC) and polymerase chain reaction (PCR) genotyping. These methods involve sophisticated instrumentations and are cumbersome and expensive.

**Results:**

In this study an innovative spectral detection method, based on the fluorescence spectra of a set of biomolecules (tyrosine, tryptophan, nicotinamide adenine dinucleotide, and flavin adenine dinucleotide and porphyrins) found in blood components is presented. An algorithm based on the spectral features of such biomolecules of blood components of 20 Thal patients (10 female and 10 male) and 18 age adjusted normal controls (4 female and 14 male) demonstrate reasonable level of classification with sensitivity and specificity values exceeding 90%.

**Conclusion:**

This new technique could be of significant value for Thal detection, diagnosis, and subsequent genetic counselling and could be adapted for use in small primary health centres.

## Background

Thalassemias (Thal) are inherited autosomal recessive blood disorders caused by α or β globin chain imbalances. Both imbalances lead to abnormal haemoglobin molecules that cause anaemia, which is a characteristic symptom of Thal. Most cases of α Thal are detected during new-borns screenings, but β Thal is not apparent until the patients are 4–6 months old.

The α globin chain is encoded by four genes, one or more of which can be mutated or missing. The affected genes cause different levels of symptoms. For example, when only one gene is abnormal, the child is a silent α Thal carrier. A patient with two missing or mutated genes is described as α Thal minor or as having the Thal α trait. Patients with three mutated Thal genes have haemoglobin H disease, and those with four missing or mutated genes have a condition known as α Thal major or hydrops fetalis [1].

The β Thal is caused by mutations in the genes that code for the β globin chain. Three forms are well known: Thal minor, Thal intermedia, and Thal major. Individuals with β Thal major usually present with severe anaemia within the first 2 years of life, poor growth, and skeletal abnormalities during infancy. Affected children require regular blood transfusions throughout their lives. The β Thal intermedia are less severe than β Thal major and therefore might require less frequent blood transfusions. Transfusion-dependent patients develop iron overloads and require chelation therapy to remove the excess iron. Bone marrow transplants can be curative for some children with β Thal major.

Thal is carried by 150 million people, representing 3% of the world’s population, and is clinically apparent in 15 million people. It is particularly associated with people of Mediterranean, Arab, and Asian origins. Certain types of Thal are more common in specific parts of the world. α Thal is common in parts of the world where malaria is or had previously been endemic. β Thal is much more common in Mediterranean countries, such as Greece, Italy, and Spain. Many Mediterranean islands, including Cyprus, Sardinia, and Malta, have significantly high incidence rates of severe β Thal, constituting a major public health problem. For example, in Cyprus, 1 in 7 individuals carry the gene, which translates to 1 in 49 marriages between carriers and 1 in 158 new born who are expected to have β Thal major [2].

On the other hand, α Thal is more common in Southeast Asia, India, the Middle East, and Africa. The carrier rates for α Thal are 1 in 7 for Cypriots, 1 in 12 for Greeks, 1 in 12 for Indians and 1 in 25 for Pakistanis. Thal is also common in the Kingdom of Saudi Arabia, particularly in the eastern and southern provinces. Premarital screening and genetic counselling centres associated with the Saudi Arabian Ministry of Health have reported a Thal prevalence rate of 18.5 cases per 1000 citizens (18 carriers and 0.5 with serious level of disease) [3].

Currently, many methods are being used to screen and diagnose Thal. The osmotic fragility test is commonly used for Thal screening, despite its low specificity. The most specific Thal diagnosis methods are haemoglobin (Hb) electrophoresis phenotyping [4] or high-performance liquid chromatography (HPLC) [5]. Other commonly used methods include polymerase chain reaction (PCR) and enzyme- linked immuno sorbent assay (ELISA) [6].

However, these techniques are expensive and cannot be used as routine laboratory tests in small hospitals or clinics. In this context, the present study describes the potential application of a spectral technique as a simple, inexpensive, and fast diagnostic protocol that could be employed in primary health centres in the Asian, Arabian, and African countries (which have highest prevalence of Thal).

The objective of the present study is to demonstrate the potential of spectral haemoglobinopathic diagnosis using well-defined Thal cases as examples. The spectra of blood cellular components and extracellular plasma could provide substantial insight into the subjects’ pathologies.

## Methods

Experiments were performed on blood plasma and red blood cell (RBC) samples from Thal patients and age-adjusted healthy controls.

Exactly 5 ml of venous blood from 18 healthy volunteers (age range: 17 to 32 years) were collected in violet sterile vials that contained the anticoagulant EDTA. The vials were gently rocked five times to adequately mix the EDTA with the whole blood, and the samples were centrifuged at 3000 rotational speed (rpm) for 15 minutes. Clear, pale, greenish-yellow plasma supernatants were obtained by such centrifugation. A total of 1.5 ml of supernatant was removed from the top layer for spectrofluorimetric analysis, leaving the buffy coat, the formed elements, and the undisturbed sediment. The blood plasma samples were subjected to synchronous fluorescence excitation spectral analyses without any other treatment.

Next, the buffy coats, which contained mostly white blood cells (WBCs) (e.g., lymphocytes), were removed and discarded, and exactly 1 ml of the thick formed elements from the bottom layer, which contained mostly RBCs, was removed to a sterile vial and mixed with 2 ml of analytical grade acetone. Proper care was taken to ensure that the formed elements did not develop lumps. After thorough mixing to enable the acetone to extract fluorophores within and around the cells, the samples were centrifuged again (3000 rpm for 15 minutes). The resulting supernatant was subjected to fluorescence emission spectra analysis at an excitation wavelength of 400 nm.

The same protocol was used to process blood samples from confirmed Thal patients (α and β major) obtained from King Khalid University Hospital, Riyadh, Saudi Arabia from where the Institutional Review Board (IRB) approval had been obtained (approval number 12/3594/IRB). The patients were informed about the investigation, and proper consent was obtained. The Thal subjects consisted of 20 Thal patients (10 female and 10 male) with a median age of 25 years (range: 17–32 years). The age and sex adjusted normal controls (N = 18) were students, researchers, and staff nurses from whom informed consents obtained.

The following side line observations were noteworthy: The ratio between the plasma and cellular component volumes in normal patient samples was approximately 1.2:1, while the same ratio in Thal patient samples was nearly 1.8:1. Control plasma was greenish- yellow in colour, while patient plasma was yellowish in colour.

### Instrumentation

The instrument used was a spectrofluorometer (PerkinElmer Luminescence LS 55) capable of collecting excitation, emission, and synchronous spectra in the 200–800 nm range. An excitation and emission slit width of 10 nm and scan speeds of 1000 nm/min were used. The samples were placed in quartz cuvettes and illuminated by a specified wavelength of light with a 10-nm spectral width and a spot size of 3 × 2 mm. The power at the point of illumination was approximately 20 μW, which was too low to induce photo bleaching. This finding was confirmed by repeating the experiment three times for each sample and observing no inter-replicate spectral differences.

Three types of spectra are measured in the field of fluorescence spectroscopy. In fluorescence emission spectra (FES), one particular wavelength is selected for the excitation of a molecule, and the fluorescence emission spectrum is obtained by rotating the emission grating over a predetermined range. The reverse is true of fluorescence excitation spectra (FXS), in which the peak emission band of a molecule is selected, and the excitation grating is rotated to scan the excitation spectra. In synchronous excitation spectra (SXS), both gratings are synchronously rotated at offsets of 40 nm or 70 nm to obtain the fluorescence excitation bands for every molecule in the predetermined range. The wavelength offset and scan range are not unique; they are determined empirically by trial and error, for a given set of experimental protocol. After analysing other offsets, including 10 nm and 30 nm, it was determined that the 70 nm offset provided excellent resolution and good contrast between the normal and Thal samples because 70 nm is the Stokes shift [7,8] of the most important biofluorophores (e.g., tryptophan) in blood plasma. Hence, all the results presented for plasma were based on the synchronous excitation spectra (SXS).

## Results

**Figure 1:**
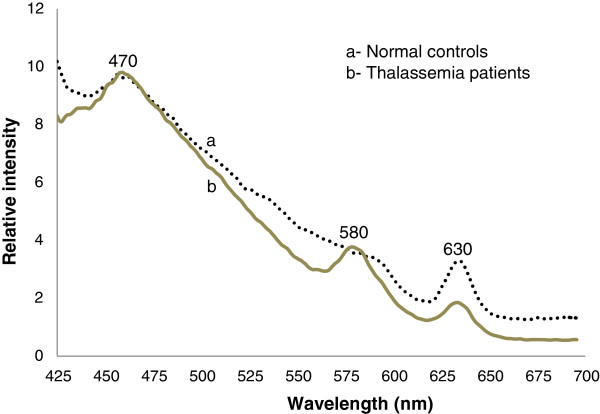
**Fluorescence emission spectra (FES) of RBC acetone extract (excitation at 400 nm). a**- Normal controls. **b**- Thalassemia patients.

**Figure 2:**
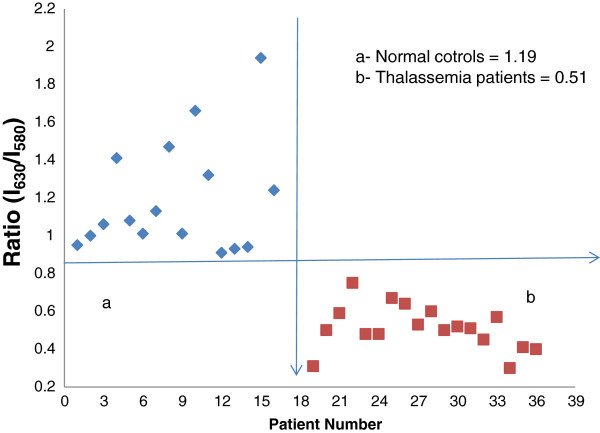
**Distribution of R = I**_**630**_/**I**_**580**_**ratios, as obtained from Figure**1**. a**- Normal controls **b**- Thalassemia patients.

Figure 1 shows the FES of the acetone extracts of blood cellular components from normal (a) and Thal samples (b), both of which were excited at 400 nm. Both had peaks at 470 nm, mostly due to the Raman acetone band in which the fluorescent bio molecules were suspended; the two additional bands at 580 nm and 630 nm represented the basic and neutral forms of porphyrin, respectively. By calculating the ratio R_1_ = I_630_/I_580_, which was the intensity ratio between the bands at 630 and at 580 nm, we obtained a value of approximately 1.2 for the normal samples and 0.5 for the Thal samples. The ratio values for these two sets are shown in Figure 2 and indicate a clear difference between the groups.

**Figure 3:**
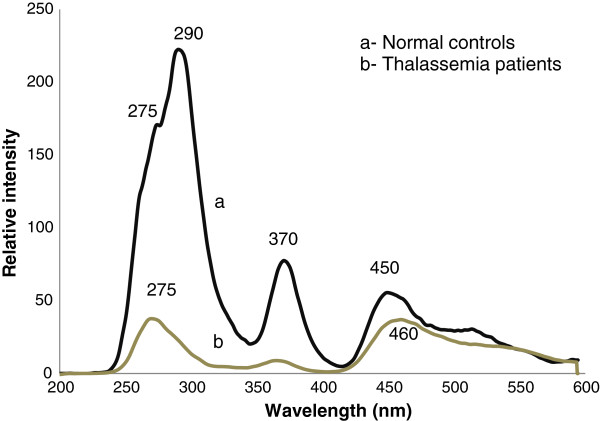
**Synchronous excitation spectra (SXS) of plasma. a**- Normal controls **b**- Thalassemia patients.

Figure 3(a) shows a typical synchronous excitation spectrum (SXS) of plasma of an age-adjusted normal control, a 25 year old female. The spectrum has three main peaks at 290 nm, 370 nm, and 450 nm. Of these, the 290 nm peak corresponded to the excitation peak of the amino acid tryptophan, the 370 nm peak corresponded to that of the coenzyme nicotinamide adenine dinucleotide (NADH), and the 450 nm peak corresponded to that of the metabolite flavin adenine dinucleotide (FAD). A shoulder was apparent at 275 nm on the shorter wavelength side of the 290 nm peak due to the amino acid tyrosine. The intensities of these peaks (in arbitrary units) were 225 for the 290 nm band, 70 for the 370 nm band, 40 for the 450 nm band, and approximately 160 for the 275 nm shoulder.

**Figure 4:**
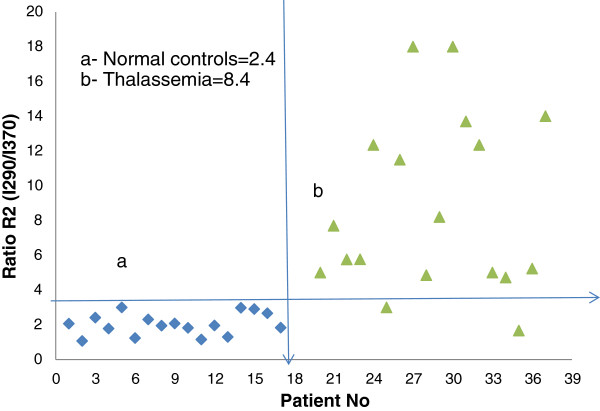
**Distribution of R**_**2**_** = I**_**290**_**/I**_**370**_**ratios, as obtained from Figure**3**. a**- Normal controls **b**- Thalassemia patients.

In contrast, a typical synchronous excitation spectrum (SXS) of plasma of a Thal patient (female, age 25) is shown in Figure 3(b). There were two major differences between the normal and Thal patient samples. The first difference was that the peaks were conspicuously out of proportion. The second was that the overall intensities were significantly lower in the Thal patients than in the normal controls. For example, the relative intensity ratio R_2_ = I_290_/I_370_ (which was the ratio of the peak intensities due to tryptophan and NADH) for the normal control was 2.4, while for the Thal patient value was 8.7. The two fluorescent biomarkers, tryptophan and NADH, were out of proportion in Thal patients, as indicated by the approximately 3.6 fold higher R_2_ value for Thal patients. The scatter plot for R_2_, shown in Figure 4, classifies the two sets quite well.

**Figure 5:**
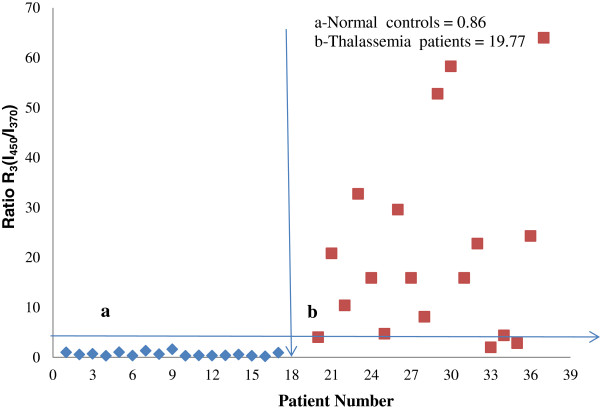
**Distribution of R**_**3**_** = I**_**450**_**/I**_**370**_**ratios, as obtained from Figure**3**. a**- Normal controls **b**- Thalassemia patients.

Similarly, R_3_, which was defined as I_450_/I_370_, or the ratio of the peak intensities at 450 nm and 370 nm due to FAD and NADH, respectively, was out of proportion in the Thal patients (Figure 3). We found that the mean of R_3_ is 0.86 for the controls and 19.7 for the Thal patients. Using this classification method, it was possible to achieve specificity and sensitivity rates exceeding 96% because the FAD levels were abnormally high in Thal patients. See Figure 5.

**Figure 6:**
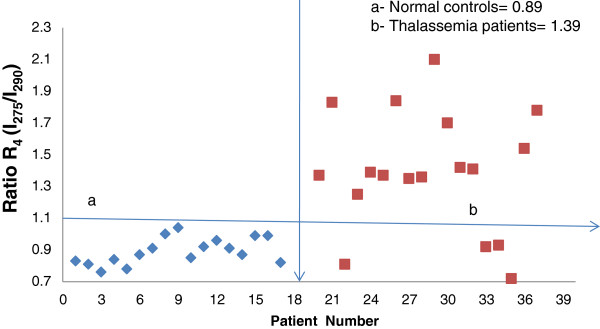
**Distribution of R**_**4**_** = I**_**275**_**/I**_**290**_**ratios, as obtained from Figure**3**. a**- Normal controls **b**- Thalassemia patients.

Another unique feature was the relative peak elevation at 275 nm compared with that at 290 nm in the Thal patients, as shown in Figure 3. The normal control samples had a clear distinct band in the UV region at 290 nm with a weak shoulder at 275 nm, but the reverse was true for the Thal patient samples. The R_4_ ratio of I_275_/I_290_ was approximately 0.8 for the normal samples but was increased to 0.9-2.1 (average: 1.39) for the Thal samples, as shown in Figure 6. Thus, the peak at 275 nm (due to tyrosine) was elevated 1.5 times in Thal patients.

**Figure 7:**
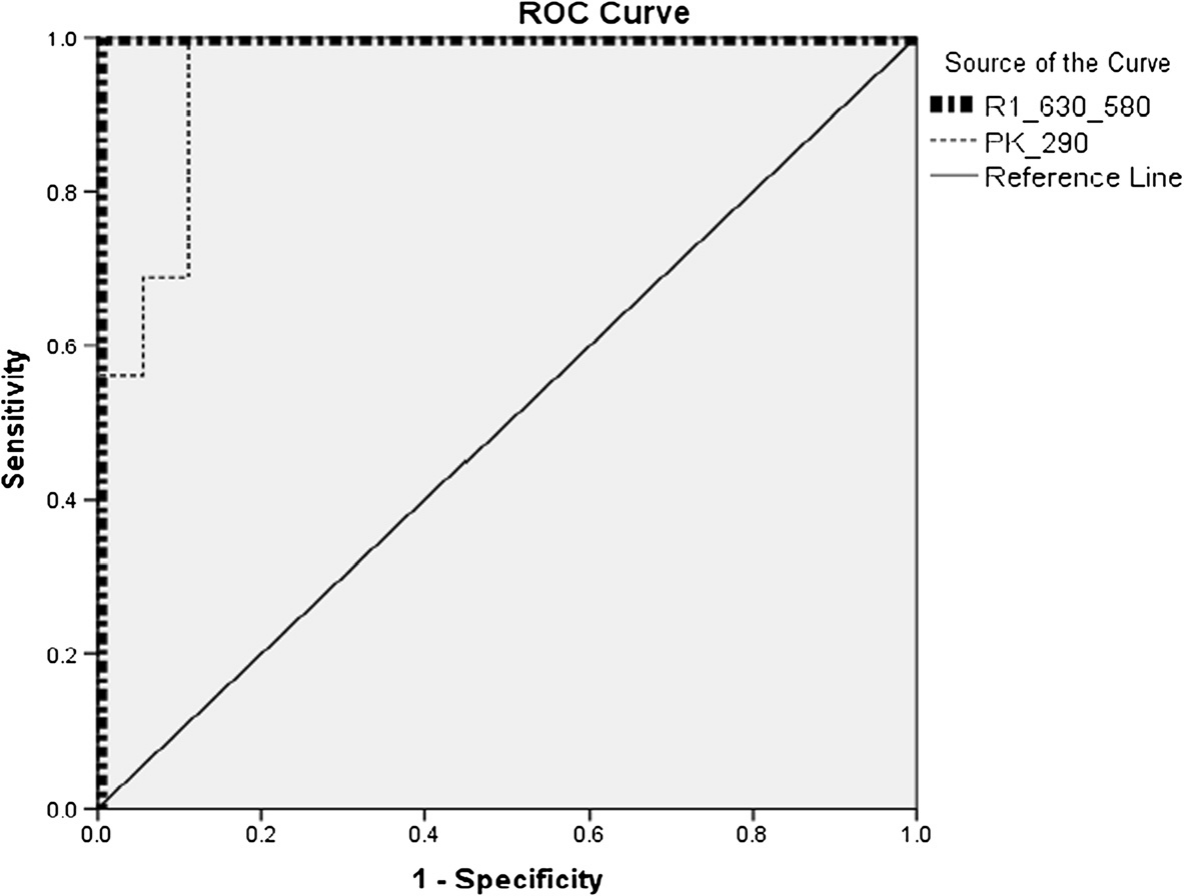
**Receiver operator curve (ROC) obtained from the statistical evaluation of the data for R**_**1**_**and the absolute intensity at 290 nm.** Note that, for R_1_, the curves run parallel to the X and Y axes.

**Figure 8:**
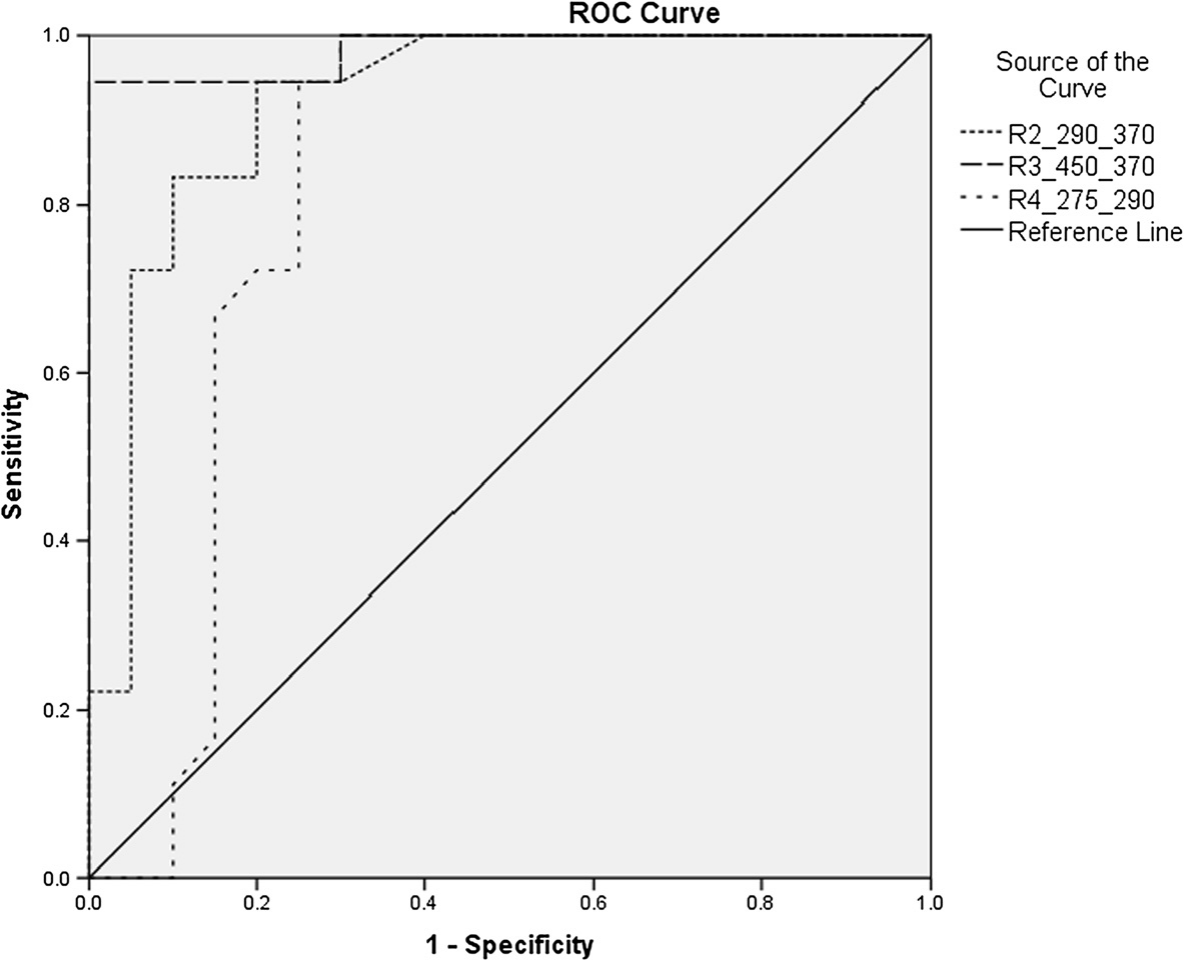
Receiver operator curve (ROC) for the other ratio parameters, obtained from the plasma SXS.

Because the present study was limited to 38 samples (20 Thal and 18 control) only a simple, basic statistical analysis was performed; the means and standard deviations for each ratio parameter are shown in Table 1 (e.g., R_1__630_580 is the ratio of the intensities at 630 and 580 nm). For each ratio, ID 1 indicates a normal sample, and 2 indicate a Thal patient sample; only one parameter, I_290_, was based on the absolute rather than the relative sample intensity. The p-value for the two-tailed test was (less than 0.05) indicating good data separation. From these data, an ROC curve was drawn for the five ratio parameters. Figure 7 shows the ROC for R_1_, which was obtained from the FES of acetone extract of RBC, and the absolute intensity parameter at 290 nm for the plasma. Figure 8 shows the ROC for other three plasma ratio parameters.

**Table 1:** **Group statistics and independent 2**-**tailed** ***t***-**test**

**Parameter**	**ID**	**N**	**Mean**	**Std. deviation**	**Std. error mean**	**2-tailed significance (P-value)**
PK_290	1.00	18	144.4833	88.16186	20.77995	
	2.00	20	24.0410	29.91528	6.68926	0.05
R1_630_580	1.00	16	1.1913	0.29768	0.07442	
	2.00	18	0.5117	0.11643	0.02744	0.06
R2_290_370	1.00	18	2.4106	1.15647	0.27258	
	2.00	20	8.7770	6.26392	1.40065	0.08
R3_450_370	1.00	18	0.8678	0.99778	0.23518	
	2.00	20	19.7755	19.16323	4.28503	0.03
R4_275_290	1.00	18	0.8994	0.10707	0.02524	
	2.00	20	1.3465	0.39160	0.08756	0.002

It is to be noted that, the ROC for the R_1,_ the runs along the two axes, indicating that this parameter is capable of classifying the samples with sensitivity and specificity levels of 100% each. The next best parameter is the R_3_ (ratio of the 450 nm and 370 nm intensities), which has sensitivity and specificity levels of 94% each (Table 2).

**Table 2:** **Sensitivity**, **specificity**, **and area under the curve**

**Test parameters**	**Area under the curve**	**Cut-off value(Threshold)**	**Sensitivity (%)**	**Specificity (%)**
R1_630_580	1.000	0.83	100	100
R2_290_370	0.922	3.5	81.3	88.9
R3_450_370	0.983	1.9	93.8	94.6
R4_275_290	0.825	1.0	87.5	77.5
PK@290	0.958	51	100	90

## Discussion

Thalassemias are blood disorders that lead to many complications, including fatigue, bone deformities, shortness of breath, growth failure, splenomegaly, and decreased life span. In developing countries, such as India and Pakistan, there are no organised programs for carrier screening or scientific counselling. In this context, the results of this study indicate that a simple spectrofluorimetric detection method could offer new prospects for inexpensive Thal detection.

A set of fluorescent blood biomarkers has been identified and quantified in Thal patients by employing synchronous excitation and fluorescence emission spectra. These spectra were compared to those of age -adjusted normal controls. The two sets showed distinctly different spectral features.

A spectral feature of cellular components is considered first.

We always measured the relative peak intensity at 630 nm (neutral porphyrin) with respect to the peak at 580 nm (basic porphyrin) as an internal reference. This ratio is dependent only on the molecules present in the sample and not on instrument factors, and it has been used extensively to detect cancer. In cancer patients, this ratio is approximately 3 or higher, while in normal controls, it is approximately 1. Thus, malignancy generally increases this ratio 3 fold [9,10]. In contrast, this ratio in Thal patients is approximately 50% of the control ratio. It is well known that the RBC content is significantly lower in Thal patients than in normal controls. In fact, the RBC content is the first test and classification factor for Thal. For all Thal cases in the present study, the RBC contents varied from 2 to 3.5 million/μL, with a mean value of 3 million/μL. In contrast, the RBC values for the normal controls, varied from 4.5 to 5.75 million/μL, with a mean value of 5 million/μL. This difference was reflected in our spectral analysis, shown in Figure 1 which is the fluorescence spectra of the cellular component acetone extracts. These findings provide direct spectroscopic evidence of the reduced RBC counts in Thal patients. As expected, we obtained a one to one correlation between the microscopic cell counts and the spectroscopic molecular diagnoses (Figures 1 and 2).

However, it was surprising to observe the effects of defective haemoglobin on the fluorescent plasma biomarkers (tyrosine, tryptophan NADH, and FAD), as shown in Figure 2 to Figure 6. Of these biomarkers, the first two are well known essential amino acids, NADH is a coenzyme, and FAD is a metabolite that is involved in many cellular redox activities. All these molecules are misregulated in Thal and cancer patients [8].

The most striking observation was the abnormal elevation in FAD levels (SXS at 450 nm) and the equally abnormal decrease in NADH levels (SXS at 370 nm); the ratio between these two biomarkers provided a diagnostic accuracy of 94%. There could be many reasons for these aberrations. For example, RBCs decays at a much faster rate in Thal patients than in normal controls due to the faulty globins, and FAD is a well-known metabolite that is involved in all cellular redox activities. In the decay of one RBC, two NADH molecules are involved, which could be the main reason for the abnormal decrease in NADH levels. Another possible reason is that NADH might transfer a significant amount of excitation energy to FAD due to the strong overlap of the NADH emission band and the FAD absorption band. Similar reductions in NADH levels and increases in FAD levels have been reported in blood and urine samples from patients with advanced and aggressive cancers, such as Hepatocellular carcinoma [8].

As shown in Figure 3, the first peak in normal plasma samples occurred at 290 nm with a shoulder at 275 nm. These peaks were reversed in Thal patient plasma samples. The ratio R_3_ = I_275_/I_290_ was approximately 0.8 for control samples and 1.4 for patient samples. Tyrosine and tryptophan are closely related essential amino acids. In Thal patients, the low oxygen levels that are caused by abnormal haemoglobin might upset the equilibrium of these amino acids.

Some essential statistics were calculated for the classifications of the normal (set 1) and Thal diseased patients (set 2), employing independent ratio parameter. R_1_ is the ratio of two types porphyrin concentrations found in cellular components; R_2_ is the ratio for FAD/NADH concentration found in plasma; R_3_ is the ratio for tyrosine/tryptophan concentration found in plasma. For each one of them, specificity and sensitivity exceeded 90%.

## Conclusion

In this preliminary spectral investigation on fluorescent biomarkers in Thal blood samples involving a limited number (N = 20) of known Thalassemia cases and age- adjusted normal controls (N = 18), distinction between the two sample sets could be achieved with an accuracy that exceeded 90%. This result is only indicative of the efficacy of spectral diagnosis. In the near future, we plan to conduct a large-scale field trial to distinguish between Thal major, minor, and intermedia, which will confer viable clinical value to this technique.

## Abbreviations

Thal: Thalassemia; EDTA: Ethylene diamine tetra acetic acid; FES: Fluorescence emission spectra; FXS: Synchronous excitation spectra; NADH: Nicotinamide adenine dinucleotide; FAD: Flavin adenine dinucleotide.

## Competing interest

The author’s declare that they have no competing interest.

## Author’s contributions

VM, MSA, and SD, made substantial contributions to the analysis and interpretations of data; Drafting of the manuscript and also responsible for overall experiments. FHA and AHA provided the biological samples. VTV did statistical analysis. KJ helped improve the text. All authors read and approved the final manuscript.
